# Readiness of Community Health Agents for COVID19

**DOI:** 10.1093/eurpub/ckac129.546

**Published:** 2022-10-25

**Authors:** A Vieira-Meyer, JMX Guimarães, APP Morais, MSA Dias, NFC Vieira, SF Farias, ALS Oliveira, CHC Meyer, MIO Vasconcelos, A Yousafzai

**Affiliations:** 1 Family Health, Oswaldo Cruz Foundation Ceará, Eusébio, Brazil; 2 Dentistry, Unichristus, Fortaleza, Brazil; 3 Nursing, Universidade Estadual do Ceará, Fortaleza, Brazil; 4 Centro de Ciências da Saúde, Universidade Estadual do Ceará, Sobral, Brazil; 5 FFOE, Universidade Federal do Ceará, Fortaleza, Brazil; 6 Public Health, Oswaldo Cruz Foundation Pernambuco, Recife, Brazil; 7 Justiça Federal do Ceará, Fortaleza, Brazil; 8 Department of Global Health and Population, Harvard University, Boston, USA

## Abstract

Brazil is one of the countries with the highest COVID19 mortality numbers. COVID19 deaths affected disproportionally different populations/communities, tending to be higher among more vulnerable ones. Brazil has a public-funded unified health system (SUS) built on the aegis of equity and social control. Its Primary Health Care (PHC) is organized by the Family Health Strategy (FHS) through Family Health Teams (FHT), which comprise a family doctor, a nurse, a dentist, nurse auxiliaries and Community Health Agents (CHAs). CHAs are individuals from the community trained to provide a range of services in the territories, including home visits, health promotion activities, and serve as liaisons between health units and communities. In this context, CHAs have the potential to play an important role in fighting the pandemic by working on contact tracing, collecting information on infected people, and providing guidance to them and the community in order to contain community transmission. However, not much is known about their readiness regarding the COVID19 pandemic in one of the Brazil's poorest regions. Thus, this study evaluated, though CHA perspective, aspects related to their preparedness for COVID19 in Brazil's northeast region. Questionnaires were applied to CHAs from 8 different municipalities - 4 capitals and 4 country-side municipalities. A total of 1935 CHAs were interviewed at their workplace in 2021. 77.8% said that they were acting in the COVID19 frontline, but only 16% referred to have received training for this function. Furthermore, only 13.7% mention to have had access to adequate individual protective equipment during their work, and 91.6% believe that they can get infected by SAR-COV-2 during their work duties. Additionally, 93.9% considered themselves a transmission vehicle due to work. Despite their potential in the fight against COVID19, CHAs did not received enough training, nor were equipped adequately during the COVID19 pandemic.

**Key messages:**

• CHAs did not received enough training, nor were equipped adequately during the COVID19 pandemic.

• Addequate trainning and work environment are essencial for proper work development.

